# Comparison of One-Year Post-Operative Evolution of Children Born of COVID-19-Positive Mothers vs. COVID-19-Negative Pregnancies Having Congenital Gastrointestinal Malformation and Having Received Proper Parenteral Nutrition during Their Hospital Stay

**DOI:** 10.3390/pediatric16040070

**Published:** 2024-09-25

**Authors:** Timea Elisabeta Brandibur, Nilima Rajpal Kundnani, Kakarla Ramakrishna, Alexandra Mederle, Aniko Maria Manea, Marioara Boia, Marius Calin Popoiu

**Affiliations:** 1 Neonatology and Puericulture Department, “Victor Babes” University of Medicine and Pharmacy of Timisoara, 300041 Timisoara, Romania; 2 Neonatology and Preterm Department, “Louis Ţurcanu” Children Emergency Hospital, 300011 Timisoara, Romania; 3 Discipline of Internal Medicine and Ambulatory Care, Prevention and Cardiovascular Recovery, Department of Cardiology, Research Centre of Timisoara Institute of Cardiovascular Diseases, “Victor Babes” University of Medicine and Pharmacy, 300041 Timisoara, Romania; 4 KL College of Pharmacy, Koneru Lakshmaiah Education Foundation, Green Fields, Vaddeswaram, Guntur 522302, Andhra Pradesh, India; 5 Faculty of General Medicine, “Victor Babes” University of Medicine and Pharmacy of Timisoara, 300041 Timisoara, Romania; 6 Department XI of Pediatric Surgery, “Victor Babes” University of Medicine and Pharmacy of Timisoara, 300041 Timisoara, Romania

**Keywords:** COVID-19, congenital gastrointestinal malformations, follow-up study

## Abstract

**Background:** The long-term effects on neonates born of COVID-19-positive pregnancies are still unclear. Congenital gastrointestinal malformations (CGIMs) often require urgent surgical intervention and antibiotic coverage. We aim to compare the health status at one-year post-surgical follow-up of cases of CGIM born of COVID-19-positive pregnancies to those of non-COVID-19 pregnancies. **Methods:** We conducted a comparative, observational study of 41 patients who underwent surgery at our hospital for congenital gastrointestinal malformations in 2022. They were initially treated with antibiotics and parenteral nutrition, which was later replaced with enteral nutrition gradually after the surgery. We then analyzed the data related to their growth and development during their 12-month follow-up visit at our outpatient clinic. We classified the children born of COVID-19-positive mothers as Group 1 (*n* = 14) and those born of mothers without COVID-19 symptoms or with unconfirmed status as Group 2 (*n* = 33). **Results:** Forty-one patients showed up for a one-year follow-up (between 11 and 13 months of life). Hence, the final Group 1 comprised 12 and Group 2 comprised 29 children. The patients were categorized based on their anatomical location. Of the cohort, 56.09% were preemies, and 43.91% were full-term newborns. We used seven parameters to evaluate both groups based on growth and developmental milestones: verbal skills, cognitive development, weight gain, height achieved, fine motor movements, gross motor movements, and social/emotional behavior. Group 1 children showed a significant decrease in height and weight compared to Group 2 children. In Group 1, 83.33% of patients were prescribed antibiotics, while only 10.34% in Group 2 were in the same situation. There were no cases of malabsorption syndrome in Group 2, but 16.66% of patients in Group 1 had it, with patients being operated on for duodenal malformations. None of the infants had necrotizing enterocolitis, post-surgical complications, or sepsis. All the children received antibiotics to prevent infection before and after surgery. No mortality was noted. **Conclusions:** In our one-year follow-up study, it was seen that even after surgical correction of congenital gastrointestinal malformations, children born of COVID-19-positive pregnancies can suffer serious growth and developmental delays, and gastrointestinal health issues might be more common. Since the long-term effects of COVID-19-positive pregnancies are not yet clear, larger cohort-based studies are required in this domain. Antibiotics destroy gut microbiota, especially in cases of gastrointestinal malformations and surgical resections. Growth and developmental milestones can not only be affected by CGIMs but also be further delayed by COVID-19 infections.

## 1. Introduction

Macronutrients play a pivotal role in the growth and development of newborns. These become more important in the recovery phase of any illness, especially in post-surgical cases. Congenital malformations are more common in preterm infants, but also, term infants are frequently found to have congenital malformations [1]. The role of macronutrients in neonates suffering from congenital gastrointestinal malformations, especially in their post-operative period, helps achieve a proper growth and development curve and prevents the deterioration of health status due to malnutrition. In addition, it also helps reduce the duration of hospital stay, overall financial burden, and morbidity and infant mortality [1,2].

In cases suffering from CGIMs, antibiotic cover is important before, during, and after surgical correction. The neonates are kept nil by mouth due to a lack of digestion capacity and to have safe anesthesia, avoiding aspiration pneumonia, especially during the surgical interventions for correction of the defect. This antibiotic cover further claims to affect the fauna of the gut of the child. This symbiosis is affected, adding up to a difficult recovery phase, which, as can be imagined, is quite tedious, as the majority of the neonates are prematurely born and have feeding difficulties due to CGIMs [3]. In normal case scenarios, prebiotics are introduced in the newborn with the breast milk fed, but in cases diagnosed with CGIM, this is not possible [4], as once the diagnosis is established, oral feedings are ceased. Human milk is known to have as one of its major components prebiotic human milk oligosaccharides (HMOs), which help a lot in the development of the immune system of the newborn [4].

COVID-19 infection causes various short- and long-term health problems. Its vulnerable groups, the elderly, pregnant females, patients suffering from autoimmune diseases, etc., were hit hardest. A study conducted by Timea B. et al. showed that children born of COVID-19-positive pregnant females were more vulnerable and might have congenital malformations and prematurity-related issues [5]. Similarly, one-fourth of the studied lot went on to have premature births in a systematic review conducted on COVID-19-positive pregnancies [6]. A study conducted in North India demonstrated a delay in achieving the 3-month development milestone in children born of COVID-19-positive pregnancies [7].

Developmental milestones are used to determine if a child is going through typical development or is having a delay in achieving these milestones. The delay can be in a certain area or in several areas of the development process. The developmental milestones are classified into social/emotional, gross and fine motor, language, and cognitive categories. The assessment of developmental disorders takes into account surveillance processes for children who are at risk and screening when asymptomatic children may be at risk of developing a disorder [7,8].

The American Academy of Pediatrics recommends screening at 9, 18, and 30 months of age. Both the Denver Developmental Screening Tests and the Age and Stage Questionnaires are used. It is mandatory to consider pathology in the neonatal period; because premature infants and newborns at risk have an increased percentage of long-term neurodevelopmental disabilities, the chronological age must be adjusted to the appropriate gestational age [9]. The World Health Organization recommends that human growth be monitored using international standards [8].

The long-term evolution of the babies born from COVID-19-positive pregnancies and having congenital malformations is still unclear. Hence, we aimed to conduct a continuation study [5] to see the one-year status of all 47 cases that were born with congenital gastrointestinal malformations and who were operated on in our hospital, out of which 14 were infants born of COVID-19-positive mothers to compare with the ones that were born of COVID-19-negative pregnancies.

## 2. Material and Methods

The same lot of 47 children that were included in our previous study [5] were taken into consideration. All 47 cases had congenital gastrointestinal malformation operations in our hospital and were given antibiotics and parenteral nutrition, which was later gradually post-operatively replaced by enteral nutrition. Data regarding their one-year status were noted upon their visit to our outpatient clinic for a regular follow-up at 12 months of life. Parents/legal guardians were asked to respond to the questions mentioned below, and their responses were recorded in Microsoft Excel sheets along with the data related to the children’s growth and development parameters. Along with this, we also collected data on if there were any admissions or emergency visits during this one year. We labeled the children (*n* = 12) born of COVID-19-positive mothers as Group 1, and Group 2 (*n* = 29) was children who were born of mothers who either did not have any COVID-19 symptoms or had symptoms but were either not tested positive or did not perform the test, and hence were considered COVID-19-negative pregnancies. There were no differences in prematurity between the 2 groups, which contributed to an equivocal and similar result.

Inclusion criteria: Gestational age (GA) ≥ 28 weeks, birth weight ≥ 1000 g, surgery for digestive tract malformations, age ≤ 7 days at admission, and complete medical history from maternity and pediatric surgery department.

Exclusion criteria: GA < 28 weeks, birth weight < 1000 g, newborns without digestive malformations, incomplete observation sheets, severe infections (sepsis or pneumonia), severe genetic malformations, post-operative deaths and loss to follow-up. These limited the cohort to 41.

### Questions Asked

Q1. Did the child frequently have diarrhea? (It was considered frequent if more than once a month.)

Q2. Did the child frequently suffer from constipation? (Considered frequent if it required regular use of medications.)

Q3. Was any kind of pro- or prebiotic treatment administered during this one year?

Q4. Was the child prescribed any antibiotics after the first discharge from the hospital until now?

Q5. Did the child suffer from any other health issues or hospital admissions than the common ones? 

Q6. Did the child have bloating, making him/her uncomfortable?

The following parameters, apart from the above responses to the questions, were analyzed: growth and development curves at one year based on weight, height, cognition, fine and gross motor movements, and social and communication skills; and malabsorption syndromes, if any. Based on the responses to the evaluation as yes—achieved desired standard milestone at one year, or no—not achieved, the percentage values were taken. Also, the presence of any post-operative complications or necrotizing enterocolitis was noted.

All children were vaccinated as per the national vaccination protocol, and had no post-vaccine unusual side effects. The birth-related and other demographic parameters can be seen from our previous study on the same lot of patients [5].

Statistical analysis: Statistical analysis was conducted using Microsoft Excel sheets in which the data were collected and percentage values were calculated. Furthermore, we performed Fisher’s exact test on the values obtained, taking into consideration the small sample size. A *p*-value of <0.05 was considered statistically significant.

Ethical approval and patient consent: The study was approved by the Ethics Committee for Scientific Research of the Emergency Hospital for Children ‘Louis Turcanu’ (approval no. 82/5 October 2023). The authors ensured that this study was carried out in accordance with the Declaration of Helsinki. Written informed consent was obtained from all patients/parents/legal guardians as a part of routine admission to our tertiary university hospital for future research and study purposes.

## 3. Results

Out of 47 children, only 41 (87.23%) showed up for a one-year follow-up (between 11 and 13 months of life). The remaining six (12.76%) children’s guardians were contacted, but some had moved to other cities and some did not respond. Hence, our final study lot comprised 41 CGIM-operated children. A total of 12 (29.26%) were in Group 1 and 29 (70.73%) comprised the final Group 2. No mortality was reported in our study lot during this one year.

The patients (*n* = 41) with operated GI malformations were further divided into four categories based on their anatomical location: malformations at the level of the esophagus (*n* = 13), pylorus (*n* = 14), duodenum (*n* = 12), and rectum (*n* = 2). From the cohort, 56.09% (*n* = 23) were preemies, and 43.91% were full-term newborns.

The demographic data of the enrolled patients are presented in Table 1.

The decision to vaccinate during pregnancy belonged to only 5 cases (12.2%), and the remaining 36 mothers (*n* = 87.8%) refused or were not informed about its possibility. At birth, newborns with digestive malformations had an Apgar score lower than 9 in 26 cases (63.41%).

**Figure 1:**
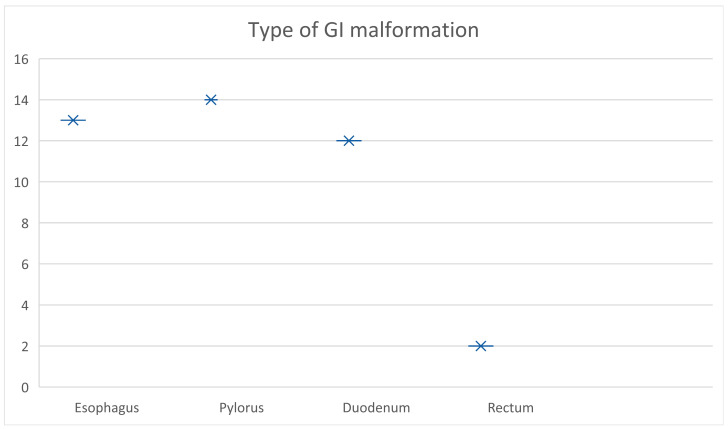
Type of GI malformation based on their anatomical location.

The type of gastrointestinal malformation was divided into four subgroups, depending on the anatomical location, in patients who presented themselves in the follow-up program (Figure 1).

The distribution of the types of GI malformations is represented in Figure 1 above, with 13 cases at the level of the esophagus (31.7%), 14 at the level of the pylorus (34.1%), 12 at the level of the duodenum (29.3%), and the remaining 2 cases (4.9%) in the rectum (Table 2).

To evaluate both groups based on their growth and developmental milestones, we included the following seven parameters corresponding to age: (1) Verbal—words spoken/communication skills; (2) Cognitive development; (3) Weight gain; (4) Height achieved; (5) Fine motor movements; (6) Gross motor movements; (7) Social/emotional behavior. We used the Fenton growth chart for preterm infants to reflect actual age instead of completed weeks to improve the growth monitoring of preterm infants.

**Figure 2:**
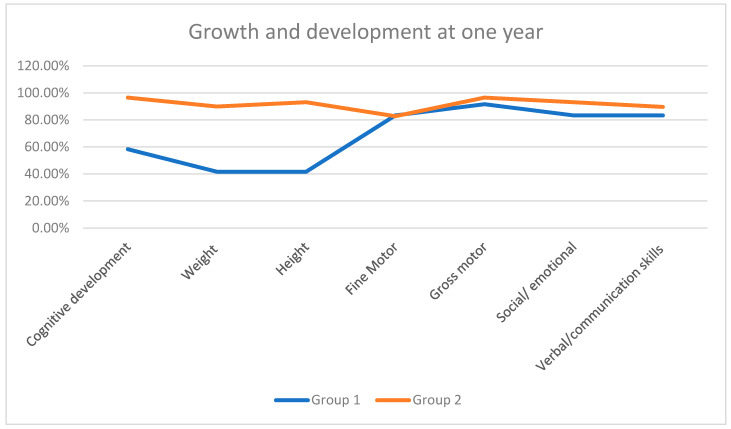
Growth and development milestones achieved in one year.

A significant decrease was noted regarding height and weight in Group 1 compared to Group 2 children. The remaining parameters, apart from cognitive development, were not found to have major setbacks (Table 3, Figure 2).

**Figure 3:**
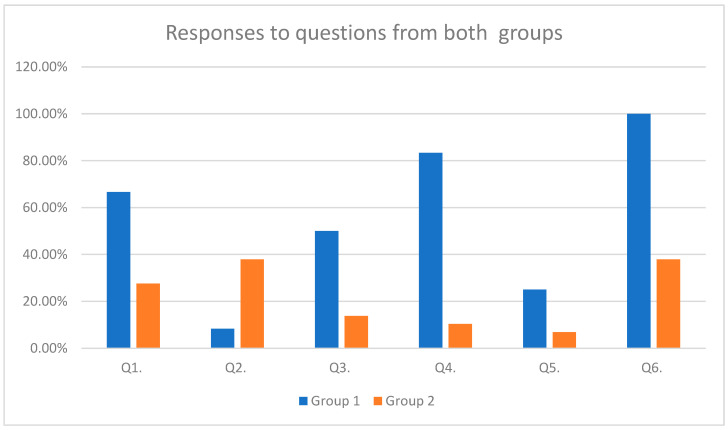
Responses to questions from both groups.

The responses of both groups to the above-mentioned questions are represented in Table 3 and Figure 3 (Table 4 and Figure 3). The percentages are based on counts of yes or no responses received.

From the Group 1 patients, 83.33% had been prescribed antibiotics for various infections during this one year, while only 10.34% were in this same situation in Group 2.

None of the patients from Group 2 presented with malabsorption syndrome. In contrast, two patients (16.66%) presented with malabsorption syndromes in Group 1, and these patients are undergoing surgery to treat duodenal malformations.

None of the infants presented with necrotizing enterocolitis, and none had any post-surgical complications or sepsis.

Furthermore, two children from Group 2 tested positive for COVID-19 at 8 and 11 months of age but had mild symptoms. They required no hospitalization.

All the children had received antibiotics pre-and post-operatively to prevent the risk of infection, according to national guidelines. Both the study lots had received parenteral nutrition with macronutrients and IV supplements during their immediate post-natal period due to CGIMs, and gradually, enteral feeds were introduced. More than 87.8% of children received breast milk, while the remaining were given formula milk. At one-year follow-up, 58.33% of patients from Group 1 were diagnosed as having mild to moderate anemia. In contrast, only five cases from group 2 (17.4%) had anemia, which was also in mild form. We did not find any relevance in the socioeconomic status of the families that could have possibly affected the study results.

## 4. Discussion

The results of our study indicate a delay in achieving normal growth and developmental milestones. The delay is seen in almost all of the CGIM-operated cases, but it is comparatively more pronounced in those born of COVID-19 pregnancies.

In a review conducted by D. Roorda et al., it was demonstrated that there were neurodevelopmental delays in children suffering from CGIMs in later life if treated successfully by surgical interventions [9]; in our studied cases, although there were delays in cognition and fine and gross motor movements, analyzing Table 3, these were not statistically significant.

In contrast, in another study, neonates suffering from various CGIMs initially suffered growth failure, but at 30 months of life, that growth had taken on a normal path [10]. Similarly, we also aim to see the progression of the cases included in our study at 2 and 5 years of life.

In the adult population, the use of pre- and probiotics has become common. Awareness has increased and protocols have been set to prescribe these along with the antibiotics. At the same time, the abuse of antibiotics has been controlled in many countries, and currently, in many countries, antibiotics are no longer available as over-the-counter drugs. And medical prescriptions are required to control their use. The use of antibiotic covers is mandatory in surgical cases, but care should be taken in the first few years of life to help the child build good immunity. The abuse of antibiotics should be controlled in neonatal intensive care units [11]. Immunity can be boosted by proper nutrition. R. Cookie et al. [12] suggested in their work a three-step approach to help treat the faltering growth of neonates due to serious and major health issues. Their plan includes establishing a normalized intake of nutrients, enriching the diet with proteins, carbohydrates, and lipids, and introducing protein-energy-enriched (PEE) formulas.

A systematic review and meta-analysis on preterm infants suffering from necrotizing enterocolitis addressed the issue of intestinal dysbiosis [13]. Similarly, studies have demonstrated that the intestinal microbiota can lead to necrotizing enterocolitis, especially in preterm infants [14]. However, no such complications were seen in our study. However, we were unable to have tests carried out to evaluate the gut microbiome due to financial constraints.

The effects of COVID-19 infection during pregnancy and its outcomes on the child in their post-natal life are still not well documented [15,16]. Nevertheless, some studies state that the incidence of neonatal pathologies was found to be on the higher side in COVID-19 pregnancies compared to non-COVID-19-pregnancy neonates [17]. In our study, it was clearly witnessed that COVID-19-pregnancy-born children were lacking in all aspects compared to non-COVID-19-pregnancy-born children and had various growth and developmental issues. Similarly, to get vaccinated or not for COVID-19 was a big dilemma worldwide. Studies stated it was safe [18], but in our study, since a very small number of vaccinated mothers were included, we fail to comment on this aspect.

### Limitations of the Study

One of the major limitations of this study is its subjective nature. We had to rely on the responses filled in by the parents or caretakers.

We did not have the financial possibility to run tests to determine the gut microbiome. Future studies with the possibility to determine gut microbiome profiling via genomic sequencing can increase the accuracy of the results.

Another limitation is the small number of study lots. Future larger studies are required with longer durations. A follow-up at one, two, five, and ten years can provide a better insight into the issue. 

Since the number of infants included in our previous study was low, we could not see a long-term follow-up in a larger cohort, as this is a follow-up study of our previous study. Recruiting new cases was not possible as the pandemic is over.

## 5. Conclusions

Congenital gastrointestinal cases should be handled with utmost care not only during their pre- and post-operative hospital admission period but also in the long term. The developmental milestones should be checked periodically and emphasis should be given to recuperating the loss as soon as possible by stimulation techniques, especially in compromised groups. The gut microbiota is destroyed whenever antibiotics are used. The prevalence of gut dysbiosis increases when the neonate is suffering from gastrointestinal malformations and has undergone surgical resections of the gastrointestinal tract. In these cases, emphasis should be given to prescribing pre- and probiotics for longer durations, as well as healthy nutrition. Similar protocols should be implemented to achieve a better quality of life in these cases. It becomes more important to keep a close watch on the long-term effects of children born of COVID-19-positive pregnancies.

## Figures and Tables

**Table 1:** Demographic data and other characteristics of the neonates.

Parameters	Percent Values
Gender	
Male	58.5% (*n* = 24)
Female	41.5% (*n* = 17)
Term infants	43.91% (*n* = 18)
Preterm infants	56.09% (*n* = 23)
Primipara	46.3% (*n* = 19)
Multipara	53.7% (*n* = 22)
Rural	51.2% (*n* = 21)
Urban	48.8% (*n* = 20)
Apgar score at 1 min below 9	63.4% (*n* = 26)
COVID-19 Status:	
Yes	36.67% (*n* = 15)
No	17.1% (*n* = 7)
Unknown	46.3% (*n* = 19)
COVID-19-vaccinated mothers	12.2% (*n* = 5)

**Table 2:** Distribution of digestive malformations according to group.

Type of Malformation	Group 1 (*n* = 12)	Group 2 (*n* = 29)
Esophagus	25% (*n* = 3)	34.48% (*n* = 10)
Pylorus	25% (*n* = 3)	37.93% (*n* = 11)
Duodenum	41.66% (*n* = 5)	20.68% (*n* = 6)
Rectum	8.33% (*n* = 1)	6.99% (*n* = 2)

**Table 3:** Growth and development milestones achieved in one year.

Parameter	Group 1 (*n* = 12)	Group 2 (*n* = 29)	*p*-Value
Verbal/communication skills	83.33% (*n* = 10)	89.65% (*n* = 26)	0.6197
Cognitive development	58.33% (*n* = 7)	96.55% (*n* = 28)	0.0053
Weight	41.66% (*n* = 5)	89.65% (*n* = 26)	0.0028
Height	41.66% (*n* = 5)	96.55% (*n* = 28)	0.0002
Fine Motor	83.33% (*n* = 10)	82.75% (*n* = 24)	1
Gross motor	91.66% (*n* = 11)	96.55% (*n* = 28)	0.5049
Social/emotional	83.33% (*n* = 10)	93.10% (*n* = 27)	0.567

**Table 4:** Responses to questions from both groups.

	Group 1 (n = 12) Response = Yes	Number of Preemies in Group 1 (*n* = 8)	Group 2 (*n* = 29) Response = Yes	Number of Preemies in Group 2 (*n* = 15)	*p*-Value
Q1. Frequent diarrhea	66.66% (*n* = 8)	62.5% (*n* = 5)	27.58% (*n* = 8)	26.66% (*n* = 4)	0.0339
Q2. Frequent constipation	8.33% (*n* = 1)	12.5% (*n* = 1)	37.93% (*n* = 11)	40% (*n* = 6)	0.076
Q3. Pre- or probiotic use	50% (*n* = 6)	50% (*n* = 4)	13.79% (*n* = 4)	13.33% (*n* = 2)	0.0402
Q4. Antibiotics use	83.33% (*n* = 10)	87.5% (*n* = 7)	10.34% (*n* = 3)	13.33% (*n* = 2)	0
Q5. Other health issues/hospital admissions	25% (*n* = 3)	25% (*n* = 2)	6.89% (*n* = 2)	6.66% (*n* = 1)	0.1394
Q6. Excessive bloating	100% (*n* = 12)	100% (*n* = 8)	37.93% (*n* = 11)	40% (*n* = 6)	0.0003

## Data Availability

Data will be made available on a valid request to the corresponding author.
